# Building bridges for empowerment and informed decision-making: A qualitative study of midwives’ reflections on how to optimise contraceptive counselling for immigrant women in Sweden

**DOI:** 10.1371/journal.pone.0340883

**Published:** 2026-02-27

**Authors:** Mia Kolak, Anette Agardh, Stefan R. Hansson, Christine Rubertsson, Maria Ekstrand Ragnar

**Affiliations:** 1 Department of Clinical Sciences, Division of Social Medicine and Global Health, Lund University, Malmö, Sweden; 2 Department of Obstetrics and Gynaecology, Skåne University Hospital (SUS), Malmö/Lund, Sweden; 3 Department of Obstetrics and Gynaecology, Institute of Clinical Sciences, Lund University, Lund, Sweden; 4 Department of Health Science, Lund University, Lund, Sweden; 5 Department of Women´s and Children´s Health, Uppsala University, Uppsala, Sweden; McGill University, CANADA

## Abstract

**Background:**

Sweden’s National Strategy for Sexual and Reproductive Health and Rights (SRHR) emphasises equitable access to contraception and abortion services. Despite this, immigrant women in Sweden experience lower contraceptive use and higher rates of unintended pregnancies and abortions compared with native-born women. Midwives, as primary providers of contraceptive counselling and prescribing, play a central role in promoting reproductive autonomy and informed choice.

**Aim:**

To explore midwives’ reflections on how to optimise contraceptive counselling for immigrant women in Sweden.

**Methods:**

Eleven focus group discussions were conducted with 50 midwives from public and private midwifery clinics in Malmö, where one-third of residents are foreign-born. Data were analysed using qualitative content analysis.

**Findings:**

The overarching theme, “Building bridges for empowerment and informed decision-making,” captured midwives’ commitment to promoting women’s autonomy while navigating systemic and cultural challenges. Five sub-themes emerged: overcoming the discrepancy between mission and reality; addressing organisational weaknesses in the care chain; enhancing knowledge and capabilities; reinforcing competence and trust in communication; and involving men without undermining women’s autonomy. Midwives highlighted structural barriers, limited resources, and time constraints that hindered person-centred and culturally sensitive care.

**Conclusion:**

Optimising counselling for immigrant women requires systemic changes, including revised funding structures, expanded multilingual resources and strengthened professional development. Addressing these barriers will enhance women's capabilities and support their reproductive autonomy, aligning with Nussbaum’s Capabilities Approach and promoting gender equality.

## Introduction

Women’s ability to make informed reproductive choices is fundamental to gender equality, consistent with the United Nations Sustainable Development Goals and the Universal Declaration of Human Rights [1,2]. The Human Development Index (HDI), developed by the United Nations Development Programme, measures health, education, and living standards worldwide, and Sweden consistently ranks among the most gender equal countries in the world [1]. Its national strategy for sexual and reproductive health and rights (SRHR), grounded in human rights, universal health coverage (UHC), and the principles of Agenda 2030, prioritises equitable access to contraception, abortion, and sexual health services [2–6].

Despite strong outcomes in gender equality and health, inequities remain in access to SRHR services [7]. Studies show that immigrant women, both globally and in Sweden, face poorer sexual and reproductive health outcomes, including lower contraceptive use and higher rates of unintended pregnancies and abortions than native-born women [8–13]. Factors such as legal status, barriers within the health system, language barriers and health literacy affect immigrant women´s access to SRHR [8–13]. About twenty percent of Sweden’s population are foreign-born, nearly half of them women [14]. In this study, the term immigrant refers to individuals who are foreign-born [15].

Sweden has a long history of midwives as primary providers of SRHR including contraceptive counselling [16]. Midwifery clinics providing antenatal, childbirth, postpartum, abortion care and contraceptive counselling were established between the 1930s and 1950s, contributing to one of the world´s lowest maternal mortality rates [17,18]. In the 1970s, a national family planning programme further strengthened SRHR by expanding contraceptive services and ensuring free access to safe and legal abortion [17,19]. Since then, midwives are the primary providers of contraceptive counselling and prescriptions, handling around 85% of all contraceptive services, including IUD insertions [18]. These services are delivered through midwifery and Youth Guidance Centres (UMO) clinics, which are widely accessible in the community and linked to hospitals for more specialised care [18]. Both public and private clinics are reimbursed equally by Sweden’s 21 regional health authorities under a system introduced in 2014 to enhance access, patient choice, and equity [20]. Today around 600 midwifery clinics operate nationwide, offering care under the principles of universal health coverage, ensuring access for all women regardless of socio-economic status or legal status, including refugees, asylum seekers, and undocumented migrants [21].

Despite Sweden's comprehensive sexual and reproductive healthcare system, offering free contraceptive counselling and subsidized contraceptives for women up to the age of 26 the unmet need for contraception has increased over the past decade, from 9% to 15–20% [22,23]. Previous research identified various reasons for not using contraceptives among women who did not wish to become pregnant, such as fear of hormones, increased bleeding concerns and preferences for non-hormonal alternatives [23,24].

For many immigrant women, their first interaction with Swedish healthcare is through a midwife, who plays a central role in promoting reproductive autonomy, especially through contraceptive counselling [18,25]. Nussbaum’s Capabilities Approach offers a valuable framework for understanding this role, as it focuses on individuals’ real opportunities to live a dignified life and exercise agency [26]. By ensuring access to reproductive healthcare and supporting informed decision-making, midwives help strengthen women’s capability (right) for practical reasoning, central to autonomy [18]. Yet, to fully realise these capabilities, structural and communicative barriers must be addressed [27].

Previous studies exploring how immigrant women and their accompanying partners experience midwife-led contraceptive counselling in Sweden highlight the importance of trust, cultural sensitivity and familiarity with the midwife’s role [28,29]. Stressful counselling sessions, described by both midwives and women, self-reported lack of knowledge about SRHR, and unfamiliarity with the Swedish health system were some of the barriers described, demonstrating a critical need to address these gaps to achieve reproductive autonomy [28,29]. In light of the needs identified, the current study aimed to explore midwives’ reflections on how to optimise contraceptive counselling for immigrant women in Sweden.

## Method

### Study design

A qualitative research design based on focus group discussions (FGDs) was chosen to gain a deeper understanding of midwives’ reflections about how contraceptive counselling can be optimised to better meet immigrant women´s needs. FGDs were chosen for their ability to encourage interpersonal dialogue and elicit shared experiences [30].

### Study setting

The study was conducted in Malmö, the third largest city in Sweden (population 360 000), known for its diversity and rapid growth. Nearly half of the population are below 35 years of age [2]. About 186 nationalities are represented and almost 50 per cent of the population are of immigrant descent, whereof about 30 per cent are foreign born, which is a larger proportion than any other city in Sweden [31,32]. Malmö, with its strategic location in southern Sweden and proximity to continental Europe, has since the 1960s been a key point of arrival for immigrants and refugees, particularly during the 2016 peak when 163,000 people, mostly from Syria, Afghanistan, and the Middle East, sought asylum in Sweden [14,31,32].

### Data collection

Data collection took place from January 2024 through March 2024. FGDs were conducted by the first author MK with co-authors AA and MER alternating as assisting researchers.

We used a purposive sampling strategy targeting midwifery clinics in Malmö, located in different parts of the city. Twelve clinics of 24 clinics were contacted; 11 agreed to participate, while one declined stating heavy workload and staff shortage. Midwives with at least one year of experience providing contraceptive counselling for immigrant women in Sweden were eligible for inclusion in the study. Clinic managers were first informed by the first author about the study by telephone and email and provided written consent. A contact midwife at each clinic acted as gatekeeper, sharing study information and coordinating appointments. Altogether, 11 FGDs were conducted, one per clinic, including public (n = 3) and private (n = 8) clinics located in both immigrant-dense areas and the city centre.

Two pilot FGDs were conducted to test and evaluate the thematic interview guide; as no revisions were needed, both were included in the analysis.

Each FGD took place in a secluded room with no other persons present other than the participants (3–6 in each group, *N* = 50), the interviewer and the assistant researcher. The FGDs were conducted until no new information emerged.

A semi-structured thematic interview guide was developed based on findings from two previous studies where immigrant women and their accompanying partners reflected upon their experiences of contraceptive counselling by midwives in Sweden [28,29]. Eight main topics were included: organisation, availability, counselling methods, knowledge, cultural and religious norms, communication, men´s role and trust. Questions were constructed for each topic. For example, under *knowledge*, one question posed was: “Immigrant women and their partners stated that they need more knowledge about contraception and SRHR, what are your reflections regarding this statement and what are your reflections about how to increase such knowledge?”

After each FGD, the moderator and assisting researcher reflected on group dynamics, the participants’ reactions to the questions, and whether new content had emerged. This allowed the researchers to reflect upon the research process and the attainment of the specific research aim. The length of the FGDs varied between 60–90 minutes (median 75 minutes). All FGDs were digitally recorded and transcribed verbatim, in both Swedish and English, by the first author.

### Ethical considerations

All participants received oral and written information about the study and provided informed consent. They were assured of confidentiality, voluntary participation and the right to withdraw at any time. Participants were assigned codes (p1–p50) to ensure anonymity. Given the group format, confidentiality could be guaranteed by the research team but not between participants. The study was approved by the Swedish Ethical Review Authority (Dnr: 2023-06848-01).

### Data analysis

The data was analysed using qualitative content analysis with an inductive approach, as described by Graneheim and Lundman, whereby categories and themes were not predefined but developed from the data through a systematic process [33]. Initially, all transcribed data was read through several times by the first and last author to gain an overall understanding of the collected data. This was followed by identifying meaning units, condensing them, coding, grouping the codes into categories and identifying emerging themes by the first author MK. Nvivo software was used as support for structuring codes [34]. By condensing the data, the process of abstraction, or lifting the text to a higher level can occur and manifest and latent content can emerge [35]. According to the analytical approach suggested by Graneheim and Lundman [33], categories represent the more manifest level by answering the question of “what” and themes represent the more underlying question of “how”. In the analysis of our data one overall theme emerged, supported by five sub-themes and ten categories. During the analytical process, discussions were held among the authors MK, AA and MER to obtain feedback and arrive at consensus about the final analytical model.

## Results

All together 11 focus group discussions were conducted, and 50 midwives participated.

The characteristics of the focus group participants are shown in Table 1.

**Table 1:** Characteristics of focus group participants.

^No. of FGD^	^Total No. Participants^	^Years of Working Experience^	^Female/Male^	^Self-identified as immigrant^	^Completed Swedish Midwifery education^
^11 FGD^	^50^ ^3-6 per group^	^Range 1–44 yrs. Median 14 yrs.^	^All female^	^8^	^All^

**Fig 1:**
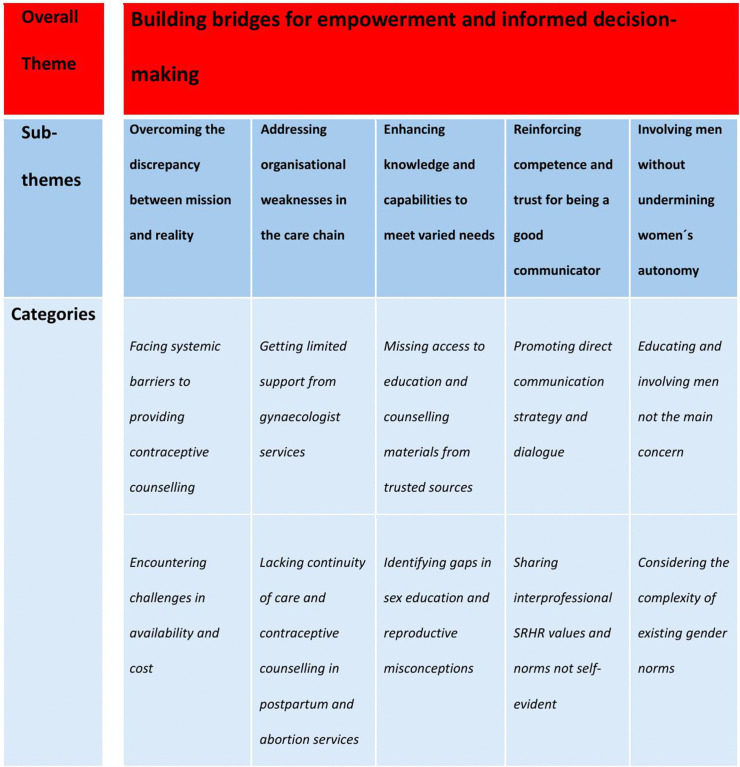
Overview of the results showing the final analytical model.

Through the data analysis one overarching theme emerged, built upon five sub-themes and ten categories (See Fig 1).

### Building bridges for empowerment and informed decision-making

The overarching theme described midwives’ overall reflections about the need for organisational changes to optimise contraceptive counselling for immigrant women and their partners. The midwives described their role as crucial in ‘building bridges’, providing essential information, supporting women’s reproductive decisions, and striving to ensure equal opportunities for all women in Sweden. At the same time, they felt constrained by systemic barriers that limited their ability to provide optimal person-centred counselling. They also emphasised the importance of understanding immigrant women’s own perspectives to strengthen empowerment and informed choice. Key challenges included creating culturally responsive dialogue and addressing immigrant men’s need for inclusion.

The text below describes the five sub-themes [in bold] and ten categories [in italics] upon which they are built. Participants are referred to by their FGD number.

#### Overcoming the discrepancy between mission and reality.

This sub-theme described midwives’ feelings of frustration about not being able to fulfil their professional responsibilities, especially towards immigrant women. Limited access to counselling appointments was viewed as a gap between the mission assigned and the reality of everyday practice. Midwives emphasised that contraceptive counselling requires time and competence to address the diverse needs of women from around the world. Yet, they felt that the system prioritised financial efficiency over quality care. They worried that immigrant women were not receiving the counselling guaranteed under Swedish law and SRHR policy and expressed concern about the long-term societal effects of reduced counselling and the gradual loss of professional competence.

##### Facing systemic barriers to providing contraceptive counselling:

According to the midwives, midwifery clinics were mainly funded by antenatal care visits, leading to lower prioritisation of contraceptive counselling. Midwives working in public clinics described an ideological difference between public and private clinics. In public clinics, care was viewed as a community service, whereas private clinics were characterised by greater autonomy in service management, although both operated within the same regional agreements. However, since all clinics, including public ones, were profit-driven, the focus on community service was somehow undermined, leading to a reduction in contraceptive counselling appointments overall. Counselling appointments were frequently cancelled or rescheduled by the staff at the clinics during holidays and other periods of staff shortage. This was described as a persistent and long existing issue at all types of clinics. Consequently, women in urgent need of emergency contraception were sometimes not seen in time, leading to unintended pregnancies. Immigrant women, especially those with limited Swedish language skills or irregular contraceptive use, were disproportionately affected.

*…”– Appointments go to pregnant women…– In the summer, we have no contraceptive visits, preferably none at all. Those appointments are cancelled. …– Yes, this is the core issue. It’s about the reimbursement”* (FGD 11).

In addition to organisational limitations, accessibility was further constrained by language barriers and administrative inefficiencies. While the system encouraged online booking and interpreter services, many women and their accompanying partners preferred visiting in person. Often, interpreter services were not booked, only to be discovered at the appointment, leading to re-bookings and delays of several weeks. Although online contraceptive counselling was available at many clinics, it was rarely used by immigrant women due to language barriers and lower health literacy. Cost-saving measures also reduced valuable frontline staff, increasing midwives’ workloads and limiting timely contraceptive care.

…”*– our clinic would not have functioned without our onsite interpreter … some women can´t speak any Swedish but they just say her name on the phone, and she can help immediately*” (FGD 7).

##### Encountering challenges in availability and cost:

Midwives noted that pharmacies sometimes withheld prescribed contraceptives due to concerns about potential interactions with other medications, which further limited access. The midwives expressed frustration, feeling that their role as prescribers was sometimes questioned by pharmacy staff, and they called for better communication and collaboration with pharmacies to avoid delays.

…”– *We’re somewhat at odds with pharmacies that still follow old guidelines…and won’t dispense the contraceptives we prescribe to breastfeeding women, which makes it hard to build trust when women return to try contraception again.* ” (FGD 10).

Also, recurrent shortages of contraceptives made it difficult for women to access their preferred methods. Additionally, the cost of contraceptives, particularly hormonal IUDs, was a significant barrier, and their large, indiscrete packaging acted as a deterrent for some women. Midwives emphasised that equal access to all contraceptive methods could not be achieved unless all contraceptives were included in the cost-protection schemes, making them affordable regardless of the chosen method.

…” – *like now, when the copper IUD is out of stock, there are no IUDs available. It’s insane… the only free contraceptive isn't even available. That could be an economic issue for women, and we can't offer it*” (FGD 8).

#### Addressing organisational weaknesses in the care chain.

This sub-theme highlighted the organisational weaknesses in the care chain that midwives perceived as obstacles to meeting the needs of immigrant women and their accompanying partners. The midwives stated that the referral system and collaboration with the women’s clinic at the hospital were vital but often inefficient, often resulting in long waiting times, sometimes up to eighteen months, before women could receive help with contraceptive issues. The midwives were worried about women “falling between the system cracks” and thereby losing their reproductive autonomy. The midwives called for more streamlined guidelines and equal opportunities to provide contraceptive care.

##### Getting limited support from gynaecologist services:

Midwives viewed gynaecologists as essential collaborators in providing specialised contraceptive counselling, but staff shortages and limited access to gynaecologists hindered comprehensive support. The obstetricians at the midwifery clinics were often overextended and lacked specific contraceptive expertise. The midwives stated that during periods of declining birth rates, midwives were assigned additional tasks to enhance women's SRHR, for example offering gynaecological exams. However, without expanded gynaecological resources, reduced referral times to specialist care at the hospital and increased prescription rights for midwives, they felt constrained in their ability to meet women's needs. Additionally, introducing charges for gynaecological services exacerbated inequalities in care access and deprioritised contraceptive counselling in an already strained system.

…”– *it is really frustrating…we have doctors who want to work with us but are restricted not to, and we have doctors who are overloaded with patients…and at the hospital there is also a lack of doctors and appointments… it is all about cost saving*” (FGD 11).

##### Lacking continuity of care and contraceptive counselling in postpartum and abortion services:

The midwives highlighted the need for a more integrated approach to contraceptive counselling and care, emphasising the importance of reaching women both postpartum and after abortion. They stressed the value of offering contraceptive options during antenatal visits and immediately postpartum, rather than waiting 6–8 weeks for the first visit as is the current practice, particularly for women who wanted to avoid pregnancy soon after childbirth. Misconceptions about the protective benefits of breastfeeding made early contraceptive counselling even more essential.

*…”-some women believe that they can´t become pregnant because they are lactating…however…it is too late waiting 8 weeks for counselling” (*FGD 3).

Similarly, the midwives identified gaps in abortion care, noting that some immigrant women avoided hospitals out of fear of being discovered by husbands or relatives. Women often turned to their trusted midwives at the clinic for emotional support, information about abortion and post-abortion contraceptive counselling. The midwives believed that offering services like home abortion care and postpartum contraceptive counselling would provide continuity of care, prevent unintended pregnancies and better address the needs of vulnerable women.

…” – *If they had an abortion or if they have a miscarriage and did not get contraceptive counselling at the hospital as they should…they should communicate it to us…so women don´t fall between the cracks”* (FGD 6).

#### Enhancing knowledge and capabilities to meet the varied SRHR needs of immigrant women.

This sub-theme described midwives’ reflections about their own knowledge, as well as how to provide more knowledge about SRHR and contraceptives, as requested by immigrant women and their partners. Midwives wanted to enhance their own capabilities in order to better meet the needs of immigrant women. Moreover, contraceptive counselling was one of the rare occasions where midwives could have a trustful dialogue with immigrant women about their sexual and reproductive health and rights and their reproductive intentions. However, midwives found it challenging to assess the immigrant women’s knowledge gaps during short and often stressful counselling visits.

##### Missing access to education and counselling materials from trusted sources:

While the midwives acknowledged having a solid foundational knowledge of contraception, they expressed a need for ongoing education in contraceptive methods and also in counselling, noting that counselling had been neglected in both their training and daily work. The midwives described a tendency to merely prescribe familiar contraceptive methods and that they lacked a specific counselling method or communication technique aligned with official guidelines. The Motivational Interviewing (MI) method introduced years ago remained as the unofficial counselling approach, with many midwives being self-taught and relying on experience. Colleague discussions were crucial for support and knowledge sharing. Attending courses during work hours was difficult, leading many midwives to pursue education in their free time. Concerns were raised regarding midwifery students receiving insufficient training in contraception, as pregnancy visits were prioritised. Regionally organised continuing education courses were offered annually but required payment, making them inaccessible to all. Information about contraceptive methods from pharmaceutical sales representatives raised concerns about trust.

…” *– We have a good education but too little opportunities to add knowledge…except for when the pharma reps come, but they don´t come as often anymore…– and there is that one course once a year…*” (FGD 4).

They also stressed the need for education on global contraceptive practices to better serve women from diverse backgrounds. With short counselling sessions being stressful, especially for women with limited knowledge, midwives called for standardised and trusted materials such as anatomical models, multilingual materials and digital apps in minority languages.

…” *– Everything today is online…but our apps for women are mainly in Swedish language… – yes, we should have apps in Arabic and Albanian for instance at our clinic …because now we are all improvising*” (FGD 7).

##### Identifying gaps in sex education and reproductive misconceptions:

The midwives described that they encountered young immigrant women and women with immigrant background, born in Sweden, lacking SRHR knowledge and expressed frustration with the school sex education. They suggested midwives should take the lead in providing SRHR education across all age groups. Collaboration between midwifery clinics, Youth Guidance Centres (UMO) and schools was seen as key to improving contraceptive use and reducing unintended pregnancies.

…” – *how can it be that growing up in Sweden and going through the Swedish school system women and girls do not have more knowledge about their own reproductive system…sometimes it is really shocking and heart-breaking*” (FGD 3).

As most immigrant women seeking contraceptive counselling at midwifery clinics were married or in partnerships, midwives perceived that the counselling needs of younger immigrant women risked being neglected. The midwives with youth centre work experience emphasised longer (60-minute) counselling sessions addressing SRHR, partner violence and honour-related issues. They believed such sessions would benefit all women and stressed the need to focus more on sexual pleasure and group discussions, similar to youth clinics’ contraceptive information in groups.

#### Reinforcing competence and trust for being a good communicator.

This sub-theme described the midwives’ reflections about their communication skills and how communication about SRHR and contraceptives could be optimised to better meet the needs described by immigrant women and their partners [28,29]. The midwives described that communication and creating trustful relationships with immigrant women were the two most fundamental tools for providing contraceptive counselling. However, how to achieve these aspects was seldom discussed within the clinics and within the midwifery profession in general. Finding ways to communicate and address often-taboo SRHR issues was challenging.

##### Promoting direct communication strategy and dialogue:

The midwives described their communication style as more direct than that of other professions, which often helped address sensitive topics but could lead to misunderstandings. They emphasised the importance of discussing taboos but noted that midwives without immigrant backgrounds tended to use a more indirect, sometimes hesitant approach. Providing extensive information supported informed choice but could also make midwives appear uncertain. Colleagues from diverse backgrounds were valued for improving communication and trust and offered important insights into immigrant women’s needs. Midwives with immigrant backgrounds were appreciated for their cultural and linguistic competence, which could provide another pathway into patients’ trust rather than differences in competence, while those without immigrant backgrounds sometimes felt constrained, fearing their words could be misinterpreted or perceived as discriminatory.

Perceived cultural norms, such as marriage prerequisites before contraception, sometimes caused hesitation and superficial counselling. Fears about being accused of racism added complexity to the interaction. Conversely, some immigrant women were less tolerant toward Swedish midwives unfamiliar with their cultures. The midwives saw themselves as bridge builders between cultures but noted that bridging SRHR, gender, and cultural norms required time, resources, and stronger institutional support.

…” *– Absolutely, racism does exist… it’s shown like you just don’t want to engage; just do the absolute basic things you have to do. You don't want to understand the patient and it's like... – Really cold treatment, absolutely…we really try to work with these things to treat all equally*” (FGD 9).

##### Sharing interprofessional SRHR values and norms not self-evident:

The midwives emphasised the importance of interprofessional collaboration and communication, particularly with Family Centres and Children’s Health Clinics (BVC), in providing holistic care for immigrant women. Collaborating with social workers and other professionals helped midwives increase access to contraceptives and SRHR services while building trust with women. However, not all clinics could collaborate with Family Centres due to financial constraints and structural issues. Moreover, successful partnerships required a communication based on shared SRHR values and norms. Finding trusted partners was sometimes difficult, with local ethnic clinics advising against contraceptives or receiving conflicting advice from imams. Cooperation with cultural doulas and interpreters was also inconsistent, as differing values could hinder collaboration. Despite these challenges, midwives agreed that having staff with immigrant backgrounds was invaluable for better understanding women's situations without judgment, reinforcing the importance of shared SRHR values.

…” *– It was with cultural doulas, and we found out from some of our women that some doulas were talking badly about contraceptives and having sex before marriage…– we were* *shocked…we had never thought of the possibility of us working together and not sharing the same core SRHR values*” (FGD 1).

#### Involving men without undermining women´s autonomy.

This sub-theme described the midwives’ responses to the presence of immigrant women´s partners during the contraceptive counselling visit. Reflecting upon men´s involvement in contraceptive counselling, the midwives stated very clearly that they were there for women, not for men. They felt honoured working with women and being their “sanctuary.” For the midwives, contraceptive counselling was primarily not about family planning within a partnership but rather about the individual woman's choice for contraception. They acknowledged that the husband sometimes could play a role, but only if the woman chose so.

##### Educating and involving men not the main concern:

Midwives expressed concern that involving husbands too closely could undermine women’s autonomy and trust during contraceptive counselling. They were reluctant to view men as patients within their professional scope, fearing this might create insecurity for women. At the same time, they acknowledged the importance of engaging and educating men to reduce opposition to contraceptives and to increase understanding of women’s anatomy. Involving men was seen as potentially supportive for women and useful for promoting shared responsibility in SRHR, particularly in discussions on preconception health.

… *“- It’s important when talking about contraceptives to not only talk about contraceptives but also about what reproductive plans they have… and you emphasise that it is not only the woman's responsibility but also the man's*” (FGD 9).

Condom use and vasectomy were considered sensitive issues linked to masculinity norms and personal choice, which midwives felt they could not easily influence—nor did they view this as part of their professional role. Although many found it challenging to involve men without compromising women’s autonomy, they sought ways to include them in SRHR discussions. Outreach to immigrant groups through Swedish For Immigrants (SFI) classes and religious institutions was regarded as essential for raising awareness, but staffing shortages and limited funding had reduced these efforts, and some midwives described engaging in such work voluntarily.

…“– *We really should discuss this thing with men… yes, but they should not be our responsibility area… we are midwives working for women, so… no, someone else could take on that assignment*” (FGD 10).

##### Considering the complexity of existing gender norms:

The midwives with limited experience of working in immigrant-dense areas described that they were initially unprepared for the complexity of providing contraceptive counselling, particularly when balancing women’s autonomy with the influence of their partners or relatives. They described difficulties in understanding women’s choices, especially when women deferred decisions regarding their reproductive autonomy to their husbands or mothers-in-law. Despite receiving detailed information on contraceptive methods, many women returned saying they needed their husband's approval or opted not to use the recommended method. Midwives also encountered women unaware of their sexual and reproductive rights in Sweden, fearing that they needed a husband's consent for contraception or abortion.

…” *– It is sometimes really difficult to understand after having informed about everything and you think that you meet in some kind of agreement and understanding…and then it all ends with “I need to go home and discuss with my husband*” (FGD 11).

The involvement of relatives or mothers-in-law, including husbands controlling menstruation cycles, further complicated women´s abilities for decision-making. The midwives found it challenging to navigate these situations, unsure whether to interpret them as reproductive repression or voluntary involvement and struggled with how to know when to intervene being afraid of being perceived as judgmental.

…”*– When I was a new midwife and new here in this neighbourhood, I wanted to make so much change for women …many years later I have realised that making change is not always what I thought it is going to be… it is more about reaching each individual making the best for her”* (FGD 3).

## Discussion

The study provides insight into midwives’ reflections about how contraceptive counselling can be optimised for immigrant women in Sweden. The overarching theme, “*Building bridges for empowerment: Needing systemic changes to optimise contraceptive counselling for immigrant women*”, indicates the midwives’ frustration about organisational barriers that hindered them in fulfilling their task. These barriers were primarily structural and located on the supply-side of the health system. Senderowicz et al. [40] argues that discussions and measurements of unmet need should distinguish between ‘demand-side unmet need’ (arising from lack of demand) and ‘supply-side unmet need’ (arising from lack of access).

In our study, supply-side barriers appeared despite the absence of formal policy or legal obstacles within the Swedish health system for providing contraceptive counselling. Midwives viewed themselves as bridge builders supporting informed SRHR choices, aligning with Nussbaum´s Capabilities Approach, which emphasises not only access to resources but also the freedom to use them meaningfully [36]. This framework clarifies how structural conditions influence women´s autonomy and supports the interpretation of the sub-themes [36].

Feeling frustrated about the discrepancy between mission and reality, the midwives described how, within the Capabilities Approach, control over one’s environment includes having the ability to influence decisions that affect one’s professional role. However, they felt constrained by funding structures that prioritise antenatal care over contraceptive counselling, limiting women’s access. A Swedish study from 2014 comparing contraceptive services in two similar cities showed reduced access during summer months, underscoring the urgent need for year-round availability of contraceptive counselling [37].

Previous research evaluating the implementation of choice reforms in primary healthcare clinics in both Europe and Sweden found a slight improvement in overall performance but also increased socioeconomic inequities in healthcare access [38,39]. Although both public and private midwifery clinics operate under regionally governed healthcare structures, there is little evidence on how equity in SRHR is affected when private providers become dominant, making it difficult to assess how the choice system influences contraceptive outcomes and resource allocation.

In addressing organisational weaknesses in the care chain, this sub-theme relates to the capabilities of health and bodily integrity [36]. Midwives described insufficient support from gynaecologists with specialised contraceptive knowledge and long referral times to hospitals with little communication between clinics and hospitals regarding whether women had received contraceptive counselling. They stressed the need for earlier contraceptive counselling after childbirth than the routine visit scheduled for 6–8 weeks postpartum, to avoid unintended pregnancies and repeated abortions. This aligns with previous research in Swedish and global settings showing that the need for and timing of effective contraception postpartum are underestimated [40–42]. Additionally, medical abortions could be offered directly through midwifery clinics if communication between hospitals and clinics was improved.

Furthermore, in Sweden, contraceptive counselling and contraception are not provided as part of routine postpartum care at the hospital [40] often explained as due to heavy workloads and lack of midwives working at the postpartum wards [43,44]. Midwifery Continuity of Care models, including early home visits postpartum, could offer an opportunity for contraceptive counselling according to women´s needs and reduce fragmentation of SRHR care [45,46]. This would reduce the gap between birth and the routine 6–8-week postpartum visit and create an earlier opportunity to discuss contraception with immigrant women in a more holistic postpartum framework. It would also allow post-abortion counselling to be provided by the same midwife, avoiding fragmentation of SRHR care.

When enhancing knowledge and capabilities to meet the varied SRHR needs of immigrant women, midwives highlighted that practical reasoning and control over one’s environment are essential capabilities that are hindered when they lack access to continuous education and resources in contraceptive counselling. Midwives emphasised the need for ongoing, targeted training to support immigrant women´s informed decision-making. Immigrant women have previously highlighted the importance of a trustful relationship and noted that meeting overworked and stressed midwives during counselling made it difficult to discuss contraceptive options in depth [47].

Globally, there is no consensus on the most effective contraceptive counselling strategy, despite the existence of multiple frameworks [48]. In line with the current findings, previous Swedish research has shown that midwives often rely on self-taught strategies to offer culturally sensitive counselling to immigrant women and highlights the need for formal education to support women regardless of their global background or life experiences [49,50]. Although several interventions across Swedish regions aim to improve contraceptive counselling for immigrant women [51,52], further investment in SRHR and training during working hours remains necessary, as emphasised in the National SRHR Strategy [53].

Our findings indicate a need to strengthen healthcare professionals’ knowledge of global SRHR issues, including contraception, to improve person-centred care and support shared decision-making, an aspect valued by immigrant women [29]. Continued investment in structured SRHR and contraceptive training for midwives, together with strengthening women´s SRHR knowledge as a key capability for informed decision-making, is therefore essential [54].

In striving to be a competent and trusted SRHR communicator, this sub-theme corresponds to Nussbaum’s affiliation capability [27]. Immigrant women have described successful counselling as grounded in mutual understanding of sexual norms, which they viewed as essential for building a non-judgmental and trustful relationship with their midwife [29]. An Australian study showed that time pressure sometimes contributed to cultural stereotyping with midwives assuming that immigrant women wanted another baby per se [55]. Immigrant men accompanying their partners in Sweden emphasised the need for sensitive communication SRHR related topics [28]. In a Swedish study of healthcare providers in women’s healthcare clinics and youth clinics, Wätterbjörk highlighted how person-centred care is shaped by two central dimensions in encounters with migrants: culture and knowledge [55].

Beyond interpersonal communication, discussing sensitive or taboo topics was perceived as challenging by both healthcare providers and patients [56,57]. In the current study, midwives reported hesitating to ask direct questions, such as about partner influence, for fear of being perceived as judgmental or racist. They also acknowledged that racism existed and even affected midwives themselves but was rarely addressed. According to Hamed et al., racism in Swedish healthcare does exist and constitutes a major barrier towards achieving equitable and responsive healthcare; however, it is not sufficiently discussed at the clinics [58].

The midwives described feeling conflicted about men´s involvement in contraceptive counselling. Building on Nussbaum’s capability of affiliation, respectful relationships should enhance rather than constrain women´s autonomy. However, previous research shows a discrepancy between women´s and men´s perceived roles: immigrant women often valued their husband´s support and believed that he knew more about the Swedish health system and contraceptives [29]. Immigrant men, however, stated that they did not have sufficient knowledge about contraceptives and SRHR and were unsure when they were expected to accompany their wives in the Swedish health system [28]. They also emphasised that they should be invited for contraceptive counselling, as they often had a decision-making role in women´s contraceptive use [28]. In response to these findings [27,28], the midwives stressed that their primary role was to empower women to make their own well-informed choices about contraceptives and SRHR and expressed concern that male participation could undermine women´s autonomy.

Rossoni et al. [59] emphasised the significant role that male partners play in contraceptive use, highlighting both their potential to inhibit women's reproductive agency, a common concern among healthcare providers, and their potential to provide support. These notions are well in line with the current results, where the midwives suggested that the concept of contraceptive counselling and how it should be provided needs to be more flexible and involve men in order to access women. In this regard, midwives suggested group counselling sessions, couple sessions including preconception health and more outreach work to meet immigrant women and men where they are in society. These activities are integrally related to midwives’ public health role but are not often provided due to lack of financing.

### Recommendations and future directions

On the basis of the current findings, the following recommendations are suggested. Funding needs to be restructured to ensure appointments for contraceptive counselling and adequate resources for comprehensive, person-centred care. Multilingual tools and online appointment access to interpreter and counselling services are needed to reduce language barriers.

Collaboration between youth clinics, midwifery clinics, pharmacies and gynaecologists need strengthening to ensure streamlined contraceptive access. Ongoing education for midwives during working hours is needed, including counselling methods and culturally sensitive counselling. Future research should evaluate the impact of regional health policies for optimised contraceptive counselling and ongoing education for midwives. More studies are needed to explore ways to involve immigrant men, such as the development of group counselling interventions for immigrant women and their partners.

### Methodological considerations

Several aspects contributed to enhancing the trustworthiness of the study [33]. In total 11 focus groups discussions were conducted. The purposive sampling of midwives with one year or more of experience of providing contraceptive counselling to immigrant women and representing various clinics distributed across Malmö reflected well the midwifery profession in Malmö, thereby strengthening the credibility of the study. However, the majority of the midwives were not immigrants, which is a potential limitation. All authors had prior SRH experience, providing a shared pre-understanding that supported reflexive awareness throughout the research process, thereby enhancing the credibility of the study. The participants were informed that the researchers moderating and conducting the FGDs were themselves midwives, which may have created a sense of trust and willingness to share and discuss openly. However, it cannot be excluded that this awareness may have constrained disclosure or introduced a sense of mistrust. In order to strengthen the study´s credibility, two midwives who participated in the FGDs reviewed the findings, as a part of a member checking process. The findings of this study are likely transferable to other midwives working with immigrant women in Sweden, as the identified themes, such as systemic barriers to contraceptive counselling and the need for ongoing education, could be relevant in similar contexts. These insights may also resonate with healthcare systems globally, facing comparable challenges.

## Conclusion

Our study findings highlight midwives’ concerns about ensuring access to contraceptive counselling for immigrant women and their partners. The barriers identified largely represent shortcomings in the health system. Addressing these systemic issues requires a restructuring of funding and healthcare priorities to ensure that midwives have the necessary resources and time to provide contraceptive counselling. Additionally, providing multilingual tools and ongoing educational development for midwives is important to help address the cultural challenges in today’s global society. By advocating for systemic reforms that emphasise equitable access to reproductive healthcare, midwives would be able to better support women’s autonomy while also reclaiming their own professional agency, aligning with Nussbaum's principle of enabling both caregivers and patients to have control over their environments.
